# Sphingomyelinase D from *Loxosceles laeta* Venom Induces the Expression of MMP7 in Human Keratinocytes: Contribution to Dermonecrosis

**DOI:** 10.1371/journal.pone.0153090

**Published:** 2016-04-14

**Authors:** Mara A. Corrêa, Cinthya K. Okamoto, Rute M. Gonçalves-de-Andrade, Carmen W. van den Berg, Denise V. Tambourgi

**Affiliations:** 1 Immunochemistry Laboratory, Butantan Institute, São Paulo, Brazil; 2 Institute of Molecular and Experimental Medicine, School of Medicine, Cardiff University, Cardiff, United Kingdom; Universidad de Costa Rica, COSTA RICA

## Abstract

Envenomation by *Loxosceles* spider is characterized by the development of dermonecrosis. In previous studies, we have demonstrated that increased expression/secretion of matrix metalloproteinases 2 and 9, induced by *Loxosceles intermedia* venom Class 2 SMases D (the main toxin in the spider venom), contribute to the development of cutaneous loxoscelism. In the present study we show that the more potent venom containing the Class 1 SMase D from *Loxosceles laeta*, in addition to increasing the expression/secretion of MMP2 and MMP9, also stimulates the expression of MMP7 (Matrilysin-1), which was associated with keratinocyte cell death. Tetracycline, a matrix metalloproteinase inhibitor, prevented cell death and reduced MMPs expression. Considering that *L*. *laeta* venom is more potent at inducing dermonecrosis than *L*. *intermedia* venom, our results suggest that MMP7 may play an important role in the severity of dermonecrosis induced by *L*. *laeta* spider venom SMase D. In addition, the inhibition of MMPs by *e*.*g*. tetracyclines may be considered for the treatment of the cutaneous loxoscelism.

## Introduction

The spiders of the genus *Loxosceles*, Sicariidae family, commonly known in the Americas as brown or recluse spiders, can induce serious local and systemic effects in humans upon envenomation [1]. *Loxosceles* envenomation can locally result in extensive tissue destruction and chronic ulcer formation which take many months to heal [2–4]. Complement mediated hemolysis, disseminated intravascular coagulation, shock and renal impairment are rare systemic reactions, but are the main causes of death associated with *Loxosceles* envenomation.

It is generally acknowledged that sphingomyelinase D (SMase D) is the main toxin in the venoms of *Loxosceles* spiders and is responsible for all effects observed in loxoscelism (reviewed in 1). Substrates of SMases D include sphingomyelin and lysophosphatidylcholine, hydrolysis of which results in the release of choline and formation of ceramide-1-phosphate and lysophosphatidic acid, both of which have the potency to activate cells [5–9].

Over 190 *Loxosceles* SMases D have now been sequenced and at the amino acid level, they display a significant level of sequence and structural similarity [10, 11]. Based on structural aspects of the enzyme a SMases D classification was proposed whereby SMases D possessing a single disulphide bridge and containing a variable loop were grouped in Class I, while SMasesD containing an additional intra-chain disulphide bridge that links a flexible loop with a catalytic loop were grouped in Class II [10–13].

As we showed previously, *L*. *laeta* venom (containing Class 1 SMases D) has stronger biological activities (haemolysis and dermonecrosis) than *L*. *intermedia* (containing Class 2 SMases D) [14]. This is reflected in the purified toxins where SMases D belonging to Class 1 have a stronger ability to induce dermonecrosis than those belonging to Class 2 [15]. Evolutionary, the majority of the Class I SMase D enzymes belong to the same clade, suggesting that they originate from a single common ancestor [10]. In the rabbit model of cutaneous loxoscelism, as well as in human keratinocytes cultures, we previously showed that *L*. *intermedia* Class 2 SMases D induced an increase in the expression of matrix metalloproteinases- 2 and 9 (MMP2, MMP9), which likely contribute to the pathology of cutaneous loxoscelism [16, 17].

MMPs are important targets for pharmacological intervention in a variety of diseases [18]. The protein synthesis inhibiting antibiotics, tetracycline and its chemically derivatives have shown also to inhibit metalloproteinases expression and function [19–22]. We previously showed that *in vitro and in vivo* tetracycline inhibited the Class 2 SMase D induced increase in expression/secretion of MMP2 and MMP9 and dermonecrosis in a rabbit model of cutaneous loxoscelism [17, 23].

Since Class 1 SMases D have a higher dermonecrosis inducing potency than the Class 2 SMase D, we aimed to investigate how the venom of *L*. *laeta*, containing Class 1 SMases D, induced keratinocyte cell death and if the dermonecrosis induced by this venom could be prevented/reduced by tetracyclines, as a potential therapeutic tool.

## Materials and Methods

### Chemicals, reagents, and buffers

Tween 20, bovine serum albumin (BSA), formalin, gelatin, Triton X-100, 3-(4,5 dimethylthiazol-2yl)-2,5 diphenyltetrazolium bromide—MTT and tetracycline were from Sigma (St Louis, MO, USA). Goat antibodies against mouse IgG (GAM), labeled with alkaline phosphatase (IgG-AP), 5-bromo-4-chloro-3-indolyl-phosphate (BCIP) and nitroblue tetrazolium (NBT), were purchased from Promega Corp. (Madison, Wisconsin, USA). MAP Human MMP Magnetic Bead Panel 2 was purchased from Millipore (Billerica, MA, USA). Brij-35 was from Fluka–BioChemika (Werdenberg, Switzerland). Mouse monoclonal antibody (MoAb) against human MMP7 was from R&D Systems (MN, USA). Tetracycline (5% in a hydrosoluble lanolin cream) was obtained from Oficinallis Pharma handle pharmacy (São Paulo, Brazil). Coomassie brilliant blue solution: 40% methanol, 10% acetic acid and 0.1% Coomassie brilliant blue. Buffers were: Saline: 0.9% NaCl; Phosphate-Buffered Saline (PBS), pH 7.2, containing 10 mM NaH_2_PO_4_, 150 mM NaCl; Zymography buffer, pH 8.3: 50 mM Tris-HCl, 200 mM NaCl, 10 mM CaCl_2_, 0,05% Brij-35.

### Spiders, venoms and SMases D

*Loxosceles* spiders were bred and maintained in house. Venom was obtained by electrostimulation by the modified method of Bucherl [7, 24]. Recombinant SMases I (Class 1) from *L*. *laeta*, P1 and P2 (Class 2) from *L*. *intermedia*, were prepared as previously described [25, 13]. Permission to access the *Loxosceles* venom (permission n° 01/2009) was provided by the Brazilian Institute of Environment and Renewable Natural Resources (IBAMA), a Brazilian Ministry of the Environment's enforcement agency.

### Animals and ethics statement

Adult New Zealand white rabbits, weighing approximately 3 Kg, were supplied by the Central Animal Breeding Facilities of the Butantan Institute, SP, Brazil. The animals were kept in a room with controlled lighting [12-h light/dark cycle] and temperature (22°C) and housed individually with water and food provided *ad libitum*. All efforts were made to minimize animal suffering and to reduce the number of animals used. The experimental animals were handled in strict accordance with the ethical principles in animal research adopted by the Brazilian Society of Animal Science and the National Brazilian Legislation no. 11.794/08. The protocol was approved by the Institutional Animal Care and Use Committee from the Butantan Institute (permission no. 840/11).

### Cell culture and maintenance

HaCaT cells (immortal human keratinocyte cell line; from Dr Fusenig, Heidelberg, Germany) was maintained in DMEM (Gibco-BRL, Gaithersburg, MD, USA), supplemented with 10% fetal bovine serum (FBS), penicillin (100 IU/mL) and streptomycin (100 IU/mL).

### Viability assay

HaCaT cells were seeded in 96-well plates at 2x10^4^ cells in 200 μl. One day before treatment, medium was removed and replaced with DMEM without FBS. Cells were, incubated with venom or SMase D in the presence or absence of tetracycline. After 24 to 72 hours, culture supernatants were collected for zymography, Western blot and Luminex analyses and cells assessed for viability by incubation with 100 μl of MTT (5 mg/mL) in PBS for 3 hours at 37°C, 5% CO_2_. Supernatants were removed and cells were incubated with 100 μl of DMSO and the absorbance was measured at 540 and 620 nm. Relative cell viability was calculated as [(sample OD _(540-620nm)_−background control OD _(540-620nm)_)/ (control OD _(540-620nm)_] x 100%.

### Gelatin zymography

Gelatinase activity was analyzed by zymography as described in detail [26]. In brief: supernatants from HaCaT cells, collected after 48 or 72 hours of incubation with SMases D or *L*. *laeta* venom, respectively, were run on a 10% polyacrylamide gel containing 0.1% gelatin. After overnight incubation at 37°C in zymography buffer, gels were stained with Coomassie brilliant blue.

### Electrophoresis and Western blot

Supernatants from the HaCaT cell cultures, collected after 48 or 72 hours of incubation with SMases D or *L*. *laeta* venom, respectively, were run on 12.5% SDS-PAGE, under non-reducing conditions and blotted onto nitrocellulose. After blocking with PBS/5% BSA membranes were incubated with MoAb against human MMP7 (1 μg/mL), followed by incubation with GAM-IgG-AP (1:7500). Blots were developed using NBT/BCIP (Promega).

### Quantification of MMP2, MMP7 and MMP9 in the supernatant of keratinocytes cell cultures

Total MMP2, MMP7 and MMP9 in supernatants samples, collected from HaCaT cell cultures, treated or not with SMases D or venom, were quantified using MAP Human MMP Magnetic Bead Panel 2 and read on Luminex (MAGPIX software).

### Dermonecrosis and treatment with tetracycline

*L*. *laeta* venom (15 μg/mL in PBS) was injected intradermally (i.d.; 200 μl) in the shaved back of rabbits. Six hours later, tetracycline cream was administered topically twice daily over a 48 hour period on the lesion area. Negative control groups consisted of animals injected with PBS and treated with tetracycline cream, while positive controls consisted of animals injected with venom and treated with hydrosoluble lanolin (vehicle). Experiments were repeated twice using 3 animals *per* group. After 48 h, the animals were euthanized by intravenous overdose of anesthetic (ketamine) and skin sections were harvested for histological examination.

### Histological analysis

Skin samples were prepared for histological analysis as previously described [16]. After staining with hematoxylin and eosin, sections were examined for the presence of epithelial necrosis, epithelial slough, dermal infiltrates, hemorrhage and level of collagen dissociation in the dermis and skin muscle fiber degeneration.

### Statistical analysis

ANOVA followed by Bonferroni test was used for statistical analysis. *P* value < 0.05 was considered significant.

## Results

### *Loxosceles laeta* venom and Class 1 SMase D induce human keratinocyte cell death

**Fig 1:**
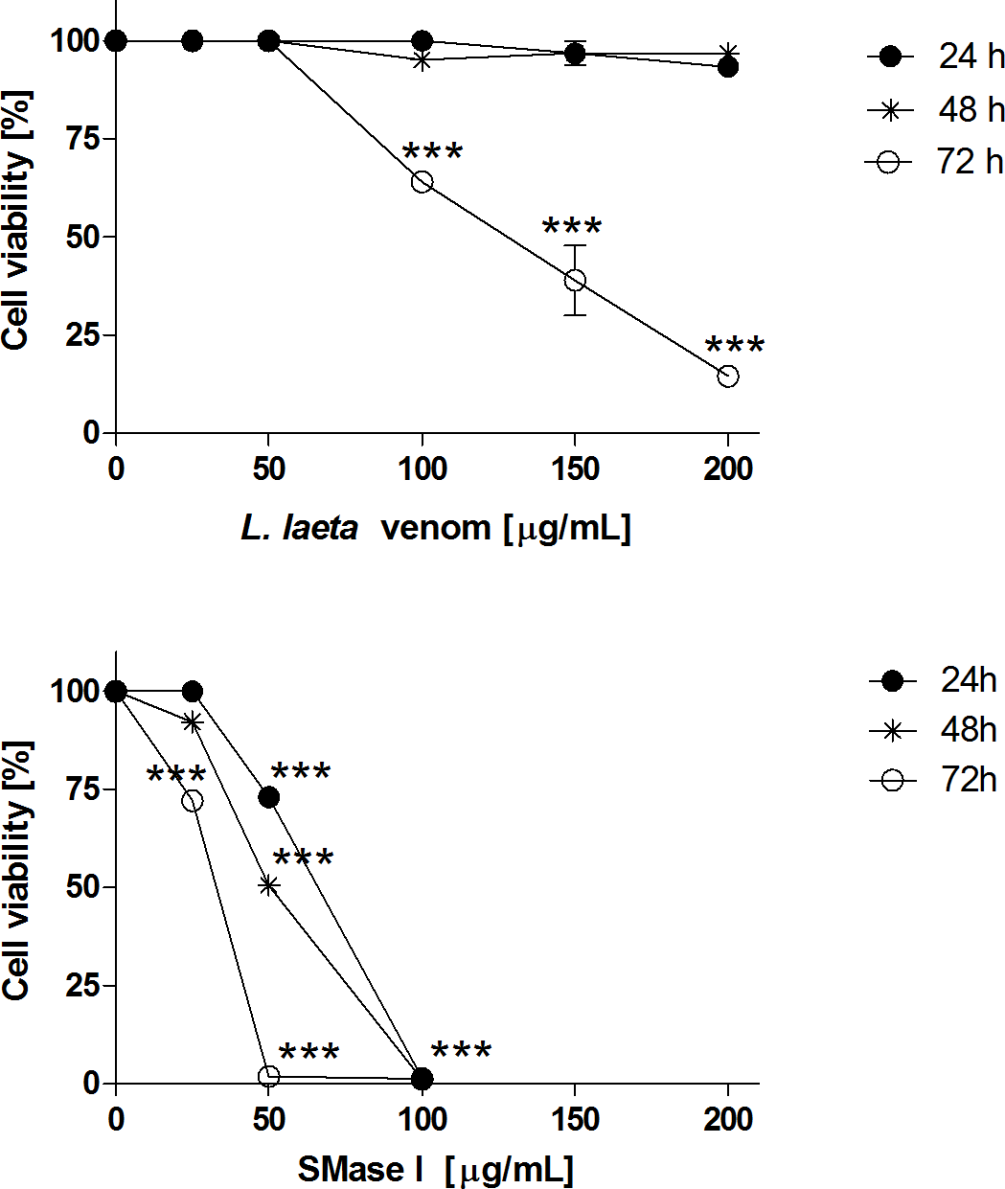
Effect of *L*. *laeta* venom and Class 1 SMase D on human keratinocytes cell viability. HaCaT cell cultures (2x10^4^ cells) were incubated with increasing concentrations (25 to 200 μg/ml) of *Loxosceles* venom **(A)** or SMase I **(B)**. After 24, 48 and 72 hours of treatment, cell viability was analyzed by MTT assay. Results are representative of three independent experiments and expressed as the mean of triplicates ± standard deviation. Significant differences (***) *P*<0.001 from control.

We previously reported that *L*. *intermedia* venom and it’s class 2 SMase D, induced cell death by apoptosis in the human keratinocyte cell line HaCaT [17]. In order to verify if Class 1 SMase D from *Loxosceles laeta* venom has the same ability, HaCaT cells were incubated with *L*. *laeta* venom or Class 1 SMase D during 24, 48 and 72 hours and the cell viability was analyzed. Fig 1 (panels A and B) shows that both, venom and Class 1 SMase D from *L*. *laeta*, induced loss of cell viability in a dose and time dependent manner. Class 1 SMase D was more potent at inducing loss of cell viability than whole *L*. *laeta* venom. At higher concentrations (100–200 μg/ml), the SMase I induced 100% of keratinocyte cell death.

### MMPs expressions in HaCaT cells supernatants

**Fig 2:**
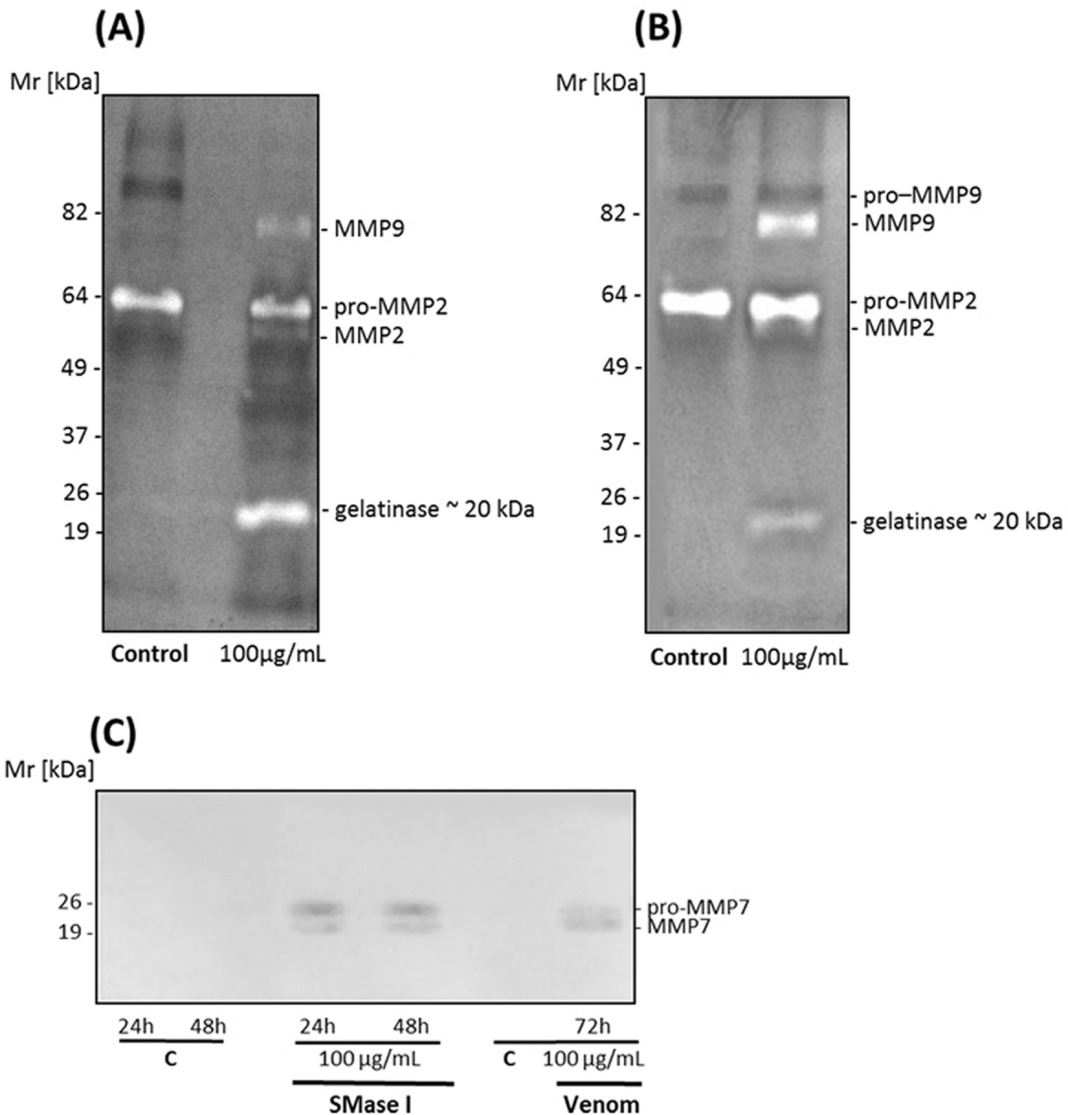
*L*. *laeta* venom and Class 1 SMase D induce the expression of matrix metaloproteinases in human keratinocytes. *Zymography analysis*: HaCaT cell culture supernatants collected after 72 hours of the treatment with *L*. *laeta* venom **(A)** or 48 hours with the SMase D Class I (SMase I) **(B)** were run on gelatin containing 10% SDS-PAGE gels under non-reducing conditions. Control supernatants were harvested after 48 and 72 hours from cells incubated with medium plus saline. **(C)** *Western blot analysis*: HaCaT cell culture supernatants, collected after 24, 48 72 hours of the treatment with medium plus saline (C), venom or SMases D from Class 1 (SMase I from *L*. *laeta*) were run on 12.5% SDS-PAGE gels, blotted and developed using MoAbs against human MMP7. Figures are representative of three independent experiments.

Since SMase I and *L*. *laeta* venom exhibit time and dose differences in cell toxicity, supernatants from HaCaT cells were investigated for MMPs expression after 72 hours of treatment with venom and 48 hours with Class 1 SMase D by gelatin zymography and western blot. Fig 2 (panels A and B) shows that venom or Class 1 SMase D from *L*. *laeta* increased expression of MMP2 (~56 kDa), as well as induced the expression of MMP9 (~80 kDa). This is similar to what we previously observed with *L*. *intermedia* venom and it’s Class 2 SMase D [17]. However, in addition a band with Mr around ~ 20 kDa was also observed in cells treated with *L*. *laeta* venom or Class 1 SMase D. In control cells, only pro-MMP2 was detected in the supernatants.

To identify the lower molecular weight band that displayed gelatinolytic activity, western blotting was carried out. Pro-MMP7 has a Mr of about 21 kDa, similar to the Mr of the band observed. Western blot analysis showed that two bands with Mr around ~ 20 kDa were detected by the anti-MMP7 monoclonal antibody, suggesting that these bands may correspond to the pro- and active forms of MMP7 (Fig 2C). In the control supernatants, pro MMP7 and MMP7 were not detected.

### Comparison of the gelatinolytic activity induced by SMases D Class 1 and Class 2

**Fig 3:**
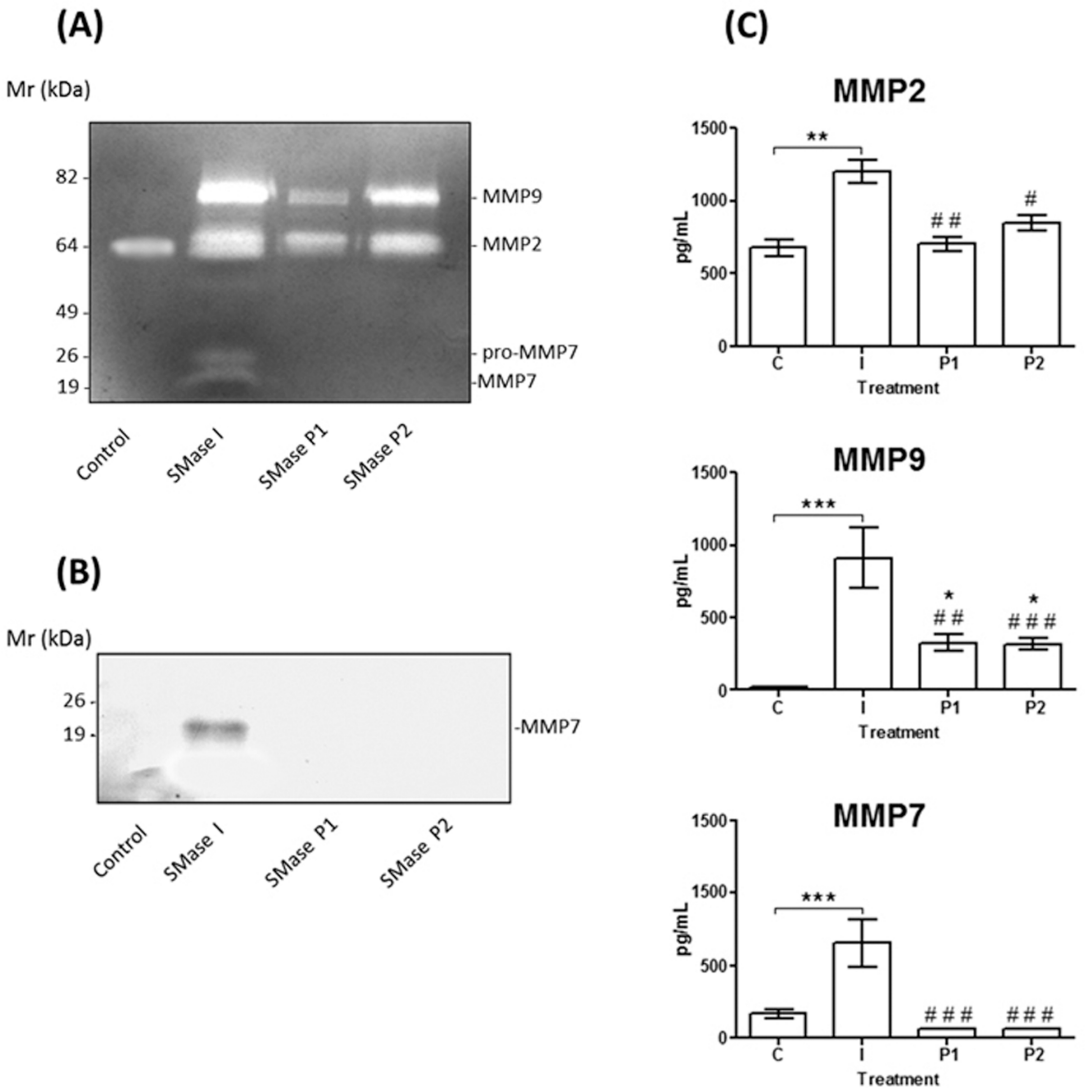
Class 1 SMase D (SMase I from *L*. *laeta*) but not SMase Class 2 induced expression of MMP7 in human keratinocytes. HaCaT cell culture supernatants, collected after 48 hours of the treatment with medium plus saline (Control) or 100 μg/mL of SMases D from Class 1 (SMase I from *L*. *laeta*) or Class 2 (P1 and P2 from *L*. *intermedia*), were subjected to zymography analysis **(A)**, Western blotting **(B)** using MoAbs against human MMP7, and Luminex assay **(C)** for human MMP2, MMP9 and MMP7. Results are representative of three independent experiments and are expressed as the mean of triplicates ± standard deviation. Significant differences (*) *P*<0.05; (**) *P*<0.01 and (***) *P*<0.001 from control or (^#^) *P*<0.05; (^##^) *P*<0.01 and (^###^) *P*<0.001 from SMases D treated cells.

Previously we only detected the presence of MMP2 and MMP9 in the supernatants obtained from human keratinocytes treated with the *L*. *intermedia* SMases D (Class 2) [17]. In order to verify if the pro-MMP7 expression was only induced by Class 1 SMases D, supernatants collected from cells treated with 100 μg/ml of *L*. *laeta* venom, SMases D from Class 1 (SMase I from *L*. *laeta*) or Class 2 (P1 and P2 from *L*. *intermedia* venom) were compared by zymography and western blot analysis using a specific MMP7 monoclonal antibody. Fig 3 (panels A and B) shows that MMP7 was detected in the supernatants of cells treated with *L*. *laeta* Class 1 SMase D but not with *L*. *intermedia* Class 2 SMases D. However, using Luminex technology, basal levels of MMP7 were detected in the supernatants of HaCaT control cells. Only Class I SMase D induced an increase in expression of MMP7, while P1 and P2 SMases, seemed to reduce MMP7 (Fig 3C). The Luminex technology also corroborated the zymography results: while SMase D, from both classes induced significant increase in MMP9 secretion, only the Class I SMase D induced a significant increase in MMP2 expression.

### Tetracycline reduces expression of MMPs and protects HaCaT from cell death

**Fig 4:**
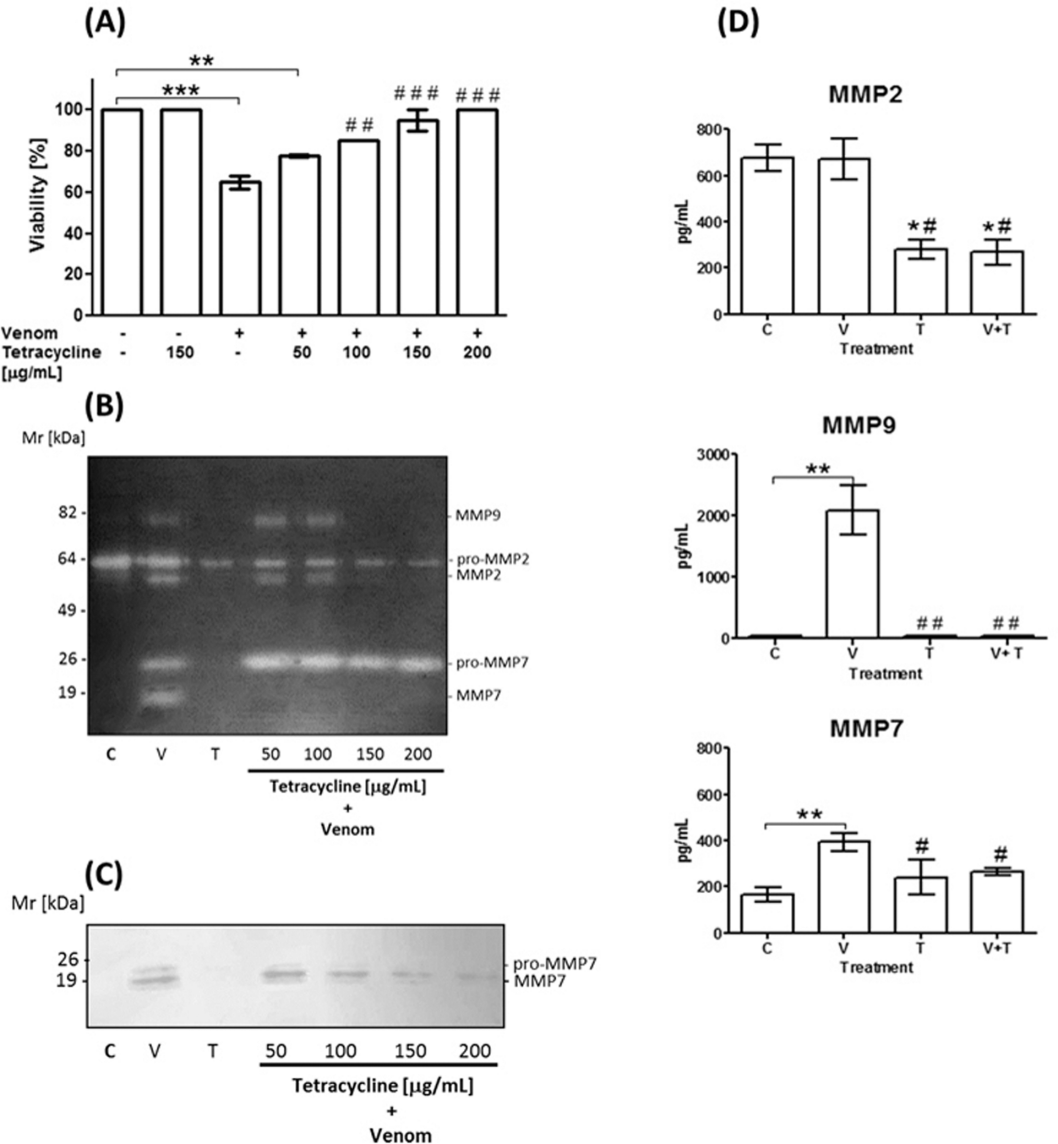
Effect of tetracycline on the cell viability and the expression of MMPs in human keratinocytes treated with *L*. *laeta* venom. HaCaT cell cultures were incubated with 100 μg/mL of *L*. *laeta* venom (V) or control medium (C) in the presence or absence of tetracycline (T). After 72 hours of treatment, cell viability was analyzed by MTT assay **(A)** and cell supernatants were analyzed by zymography **(B),** western blotting **(C)** and Luminex assay (tetracycline: 150 μg/mL) **(D).** Results are representative of three independent experiments and are expressed as the mean of triplicates ± standard deviation. Significant differences (*) *P*<0.05; (**) *P*<0.01 and (***) *P*<0.001 from control or (^#^) *P*<0.05; (^##^) *P*<0.01 and (^###^) *P*<0.001 from venom treated cells.

We have previously shown that cell death through apoptosis induced by *L*. *intermedia* venom and Class 2 SMases D in HaCaT keratinocytes could be prevented by metalloproteinase inhibitors, such as tetracycline [17]. In order to verify if tetracycline was also effective at inhibiting biological effects of Class 1 SMaseD in *L*. *laeta* venom, HaCaT cells were incubated with *L*. *laeta* venom in the presence of various concentrations of tetracycline. Indeed, tetracycline dose dependently protected HaCaT cells from venom induced cell death and complete protection was obtained at 200 μg/mL tetracycline (Fig 4A). Tetracycline did not affect HaCaT cell viability of control cells.

Effects on metalloproteinases expression/secretion by tetracycline were evaluated by zymography, western blotting and luminex technology. Fig 4 (panels B, C, D) shows that tetracycline abolished the conversion of secreted pro-MMP7 into active MMP7. Western blot showed that at higher concentrations tetracycline also reduced the secretion of pro-MMP7. In addition, tetracycline significantly inhibited MMP2 and MMP9 expression induced by *L*. *laeta* venom treatment.

### Tetracyclines reduces the dermonecrotic lesion

**Fig 5:**
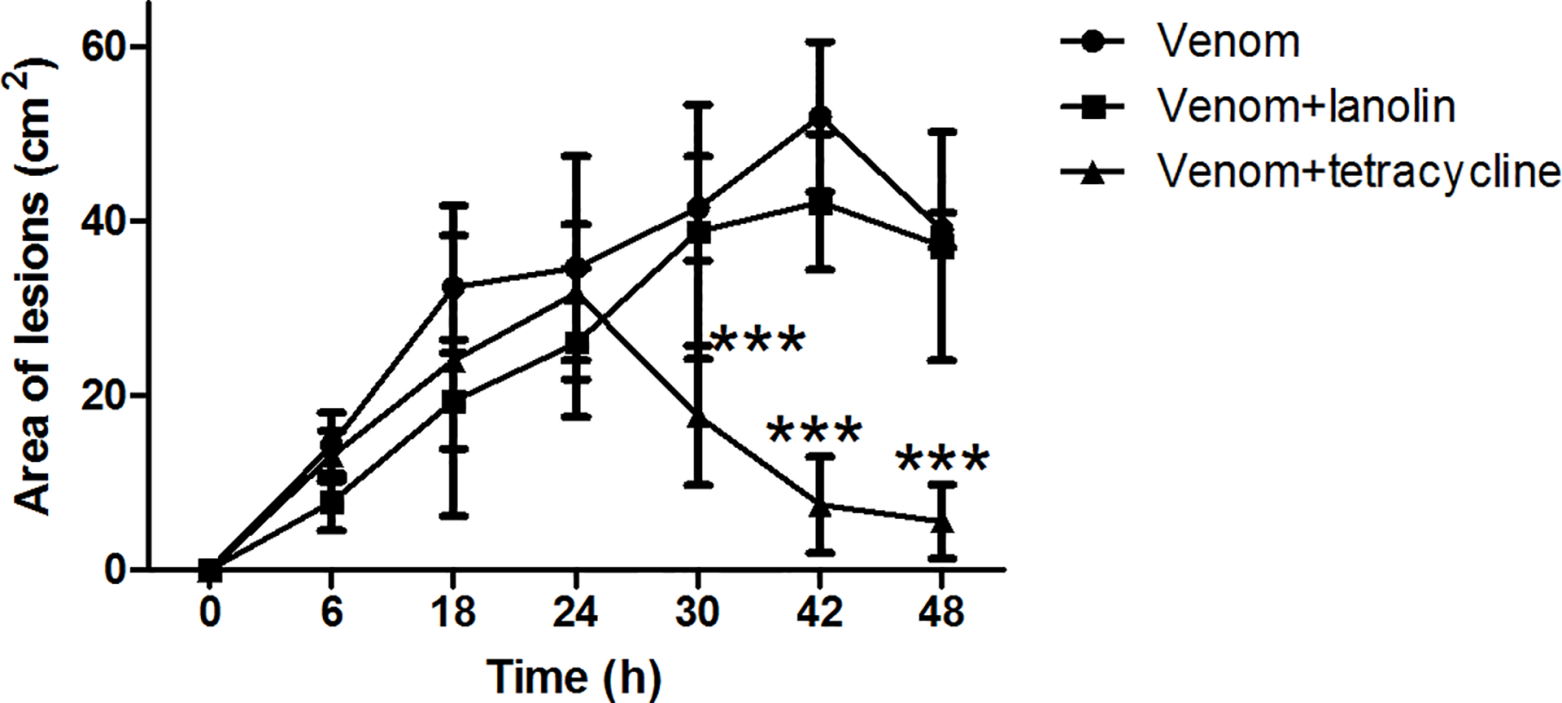
Effect of tetracyclines on dermonecrotic lesions. **(A)** Adult rabbits were injected intradermally with 3 μg of venom from *L*. *laeta* spiders. After 6 h the animals were treated topically (black triangle) with a cream containing 5% tetracycline, twice a day during 48 hours. Control animals were inoculated with venom and either not treated (black circle) or treated with lanolin cream alone (black square) 6 hours after venom injection. Results are representative for two independent experiments and are expressed as the mean of triplicates ± SD. The asterisks indicate values statistically different (***) P<0.001 from the controls, *i*.*e*., non-treated or lanolin cream treated venom injected animals.

The ability of tetracycline to inhibit *L*. *laeta* venom induced dermonecrosis was assessed by topical administration of tetracycline containing cream at the sites of venom injection (Fig 5). Control animals, injected with venom but not treated with tetracycline cream, developed within a few hours the typical loxoscelic lesions, characterised by edema, erythema and mild tenderness. 24 h later, necrosis with gravitational spread and scar had developed and the lesion size increased over 48 h. Topical administration of tetracycline cream twice daily over 48 hours significantly reduced the venom induced dermonecrotic lesion while vehicle only (lanolin) did not have an effect.

### Histological analysis of skin lesion

**Fig 6:**
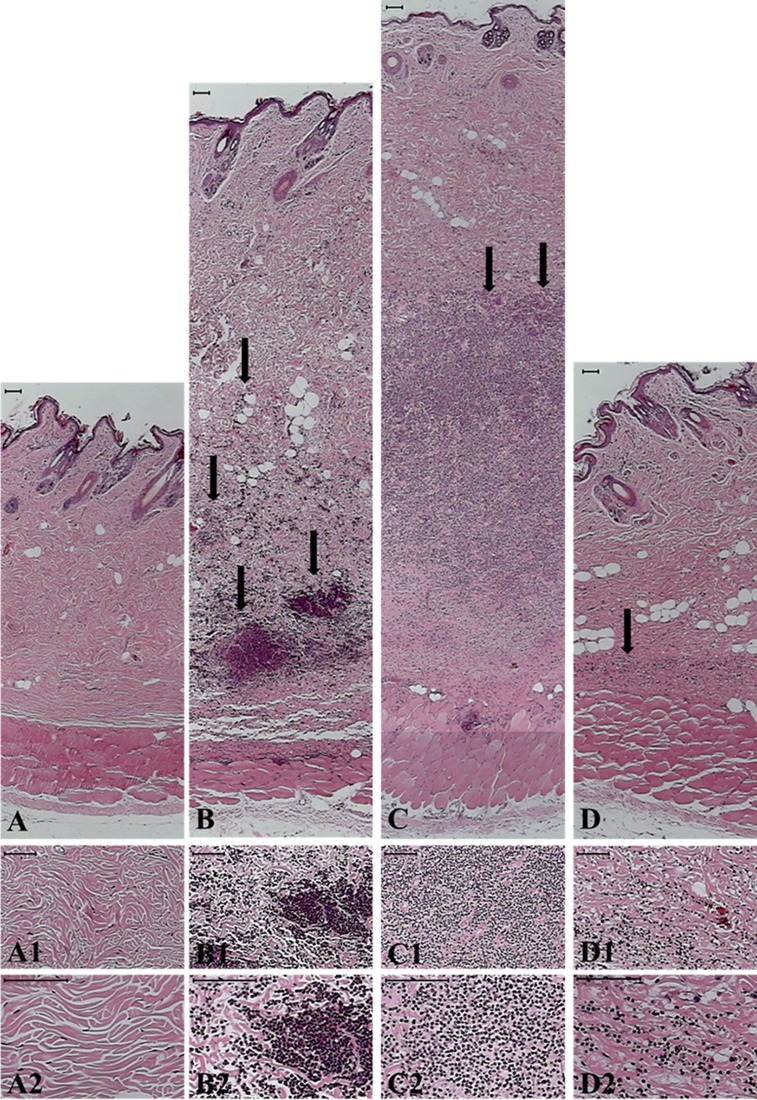
Histological analysis of the dermonecrotic lesion induced by *L*. *laeta* venom after tetracycline treatment. Rabbits were injected with 3 μg/mL of *L*. *laeta* venom and treated, twice a day during 48 hours, with tetracycline containing cream. Control sites were injected with an equal volume of PBS. Panels correspond to the panoramic view of skin sections from rabbits injected with PBS **(A),** *L*. *laeta* venom **(B),** *L*. *laeta* venom and treated with lanolin cream vehicle **(C),** *L*. *laeta* venom and treated with lanolin cream containing tetracycline **(D)**. Arrows indicate areas of leukocyte infiltration. Panels 1–2 show details of collagenous area of the dermis of the same sections. Black bars at the top of each panel indicate 100 μm.

Histopathological analysis, of rabbit skin 48 h after intradermal *L*. *laeta* venom injection, showed a normal epidermis, dissociation of the collagen fibers due to the edema and deep intradermal intense neutrophil infiltration (Fig 6B), degeneration of muscle fibers, and discrete neutrophil infiltration in the muscle layer. Topical treatment with tetracycline after venom injection largely prevented edema and neutrophil infiltration and neutrophil invasion into the muscle layer (Fig 6D). Lanolin (vehicle) treatment did not prevent the edema and neutrophil infiltration, although the latter was more dispersed, possibly caused by the physical action of the massaging of the lanolin in the affected area. (Fig 6C). Skins of rabbits subjected to the venom plus tetracycline treatment were histologically similar to that of PBS-inoculated animals (Fig 6, panels A and D) and far lower infiltration of neutrophils than in the venom injected animals was observed (Fig 6, panels B and D).

## Discussion

Dermonecrosis is a well-documented consequence of envenomation by *Loxosceles* spiders in humans. SMase D is the principle enzyme in *Loxosceles* venom responsible for the major pathological effects. In human keratinocytes *Loxosceles intermedia* Class 2 SMases D induced the expression of matrix metalloproteinases-2 and 9 (MMP2, MMP9), concomitant with the cutaneous loxoscelism lesion, as we previously reported [17].

In Brazil, medically important species of *Loxosceles* include *L*. *laeta* and *L*. *intermedia*, *L*. *laeta* being the most potent in inducing dermonecrosis [14]. We show here that *L*. *laeta* venom and it’s recombinant Class 1 SMase D, in addition to an increased expression of MMP2 and -9 as previously shown for Class 2 SMase D, also induced the expression of a molecule with gelatinolytic activity with Mr around 20 kDa in the HaCaT keratinocytic cell line (Figs 2 and 3). Using western blotting and MMP7 specific Luminex assay, we identified this molecule as MMP7/Matrilysin-1. The increase of MMPs expression/secretion coincided with a reduction of cell viability. The increase in expression of MMP2 and induction of expression/activation of MMP7 and MMP9 was inhibited by tetracycline. While tetracycline only slightly inhibited the induction of pro-MMP7, it largely prevented the conversion into mature MMP7. *In vivo*, tetracycline inhibited the development of the lesion and enhances recovery. Concomitantly, it reduced the tissue destruction and the influx of neutrophils.

SMases D displays a typical TIM (α/β)_8_-barrel fold and its active-site cleft is surrounded by the catalytic loop, variable loop, flexible loop and other short hydrophobic loops. While Class 1 SMases D contain one disulphide bridge, SMases D Class 2 contain an additional disulphide bridge which connects the catalytic loop to flexible loop, diminishes the active-site volume and also alters the inherent flexibility exhibited by the flexible loop [10–12]. This difference may explain the differences in potency of the *L*. *laeta* class 1 SMases D (Class I) and the *L*. *intermedia* (class 2) SMases D, resulting in a high expression of MMP7, MMP2 and MMP9, compared to class 1 SMases D that induced MMP9 and not MMP7.

The observation that SMase D can induce the high expression of MMP7 is novel. MMP7/matrilysin, secreted as a 28 kDa proenzyme and becomes activated after enzymatic cleavage of its N-terminal 9-kDa prodomain [27, 28]. MMP7 belongs to the group of matrilysins, which also includes MMP-26. Matrilysins are responsible for degrading extracellular matrix components and MMP7 substrates include elastin, proteoglycans, type IV collagen, fibronectin, and nidogen [29–31]. MMP7 expression in keratinocytes is increased in pro-inflammatory conditions *e*.*g*. such as caused by tissue trauma resulting in acute and chronic wounds [32], autoimmune diseases such as lupus [33] and by ultraviolet light in HaCaT cells [34] and is also increased in skin carcinomas [35, 36].

Non-healing chronic wounds are characterised by uncontrolled proteolytic tissue destruction in which MMPs play an important role. In addition to inhibition of MMP expression, tetracycline also inhibits the activation from pro-MMPs to mature MMPs, as well as the enzymatic activity of MMPs [37]. Previously, we have shown that the increased expression of MMP2, MMP9, keratinocyte cell death and the *in vivo* dermonecrotic lesion formation induced by *L*. *intermedia* venom could be prevented by the use of tetracycline [17, 23]. We show here that tetracycline can also modulate the action of *L*. *laeta* venom *in vitro* and *in vivo*. And as shown here for the first time, this inhibitor also prevents the expression/secretion and activation of MMP7 induced by *L*. *laeta* venom in HaCaT cells (Fig 4).

Interestingly, MMP7 has been reported to cleave pro-forms of MMP2, MMP9 [38] and activate ADAM28 [39]. Furthermore, Li and collaborators showed that MMP7 is needed for transepithelial neutrophil migration in acute lung injury. In its absence, neutrophils cannot cross the lung epithelium, and accumulate in the interstitial space between the capillaries and the epithelium, which demonstrates that MMP7 is needed for directed neutrophil migration in injury [40, 41]. Neutrophils are a major contributor to dermonecrosis after envenomation [42, 16, 23] and considering the importance of MMP7 for neutrophil migration, our observation that the class 1 SMase D containing *L*. *laeta* venom induced MMP7 high secretion, may explain why it is more potent than the Class 2 SMases D in inducing dermonecrosis. Furthermore, our observation that tetracycline inhibits the activation and expression of MMP7 (Fig 4) may explain our additional observation that tetracycline inhibits the infiltration of neutrophils after venom injection (Figs 5 and 6).

Recently, lesion formation induced by *N*. *nomurai* jellyfish envenomation was found also to be associated with increased MMP2 and MMP9 expression; this MMPs and lesion formation were also inhibited by tetracycline [43]. Although the substance responsible for induction of these lesions and MMP-expression have not been identified, our and Kang’s observations suggest that tetracycline therapy may be useful in the treatment of dermonecrotic lesions of various origins. In addition, due to its antimicrobial action, tetracycline treatment of cutaneous loxoscelism has the added advantage that it may prevent or treat concurrent wound infection.

In conclusion, we show here that *Loxosceles laeta* venom and its Class 1 SMase D induce and/or enhance the expression of matrix metalloproteinases (MMP2, -7 and -9) and cause cell death. The induction of MMP7 in high quantities, as a consequence of *L*. *laeta* venom and Class I SMase D action on HaCaT, may explain the enhanced severity of the envenomation induced by *L*. *laeta* compared to that of *L*. *intermedia*. Reduction of dermonecrotic lesion and inhibition of MMPs using tetracycline as shown here may be a suitable therapeutic intervention after envenomation by different *Loxosceles* species.
